# *Aspergillus fumigatus* in a fine needle aspiration of a cavitary lung lesion

**DOI:** 10.1002/ccr3.1876

**Published:** 2018-11-05

**Authors:** Wencheng Li, Avinash K. Shetty, Ziyan T. Salih

**Affiliations:** ^1^ Pathology Wake Forest Baptist Medical Center Winston‐Salem North Carolina; ^2^ Pediatrics & Infectious Disease Wake Forest Baptist Medical Center Winston‐Salem North Carolina

**Keywords:** *Aspergillus fumigatus*, fine needle aspiration, pulmonary cavitary lesion

## Abstract

The diagnostic utility of fine needle aspiration cytology to detect a wide variety of opportunistic pulmonary infections in an immunocompromised host has been studied. Fine needle aspiration cytology techniques are safer, cost‐effective and provide rapid results.

A 64‐year‐old male presented with chills and mild dyspnea on exertion after completing four cycles of chemotherapy for stage IIA adenocarcinoma of the lung. Five months prior to presentation, he underwent left upper lobe lobectomy. He denied cough, chest pain, or fever. On examination, he was afebrile with normal vital signs. Chest auscultation revealed normal breath sounds. The rest of the physical examination was unremarkable.

Laboratory tests showed mild anemia with normal values of white blood cell count and platelet count. A chest computed tomography (CT) scan revealed a new 2.2 × 1.8 cm, thick‐walled cavitary lesion in the superior aspect of the left lower lobe and mediastinal lymphadenopathy. Serum galactomannan antigen test was negative. A diagnostic CT guided percutaneous left lower lobe fine needle aspirate (FNA) was performed. By standard procedures, paired aspirate smears were obtained and stained with Diff‐Quik (Figure 1) and Papanicolaou stains (Figure 2). Cell block material was stained by hematoxylin‐eosin (Figure 3) and Gomori methenamine silver (GMS) stains (Figure 4). Cultures for bacteria, fungi, and mycobacteria were also obtained.

**Figure 1:**
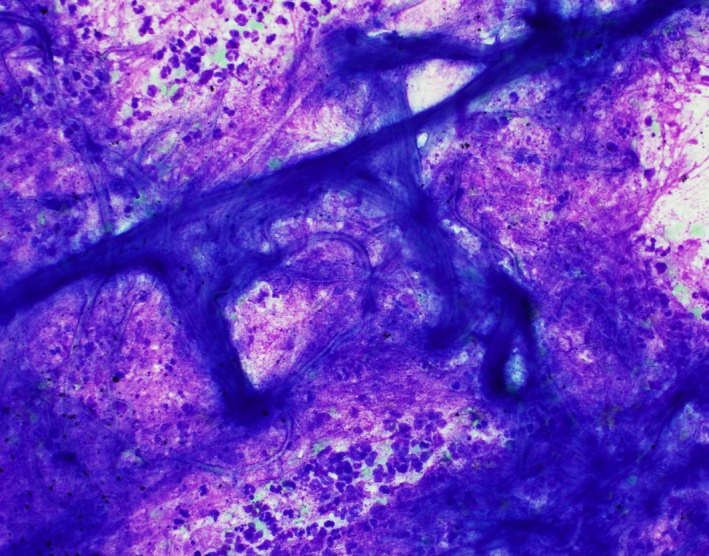
Smear preparation evaluated onsite at the time of the fine needle aspirate (FNA) procedure demonstrate entangled fungal hyphae within a background of necro‐inflammatory debris (Diff‐Quick stain)

**Figure 2:**
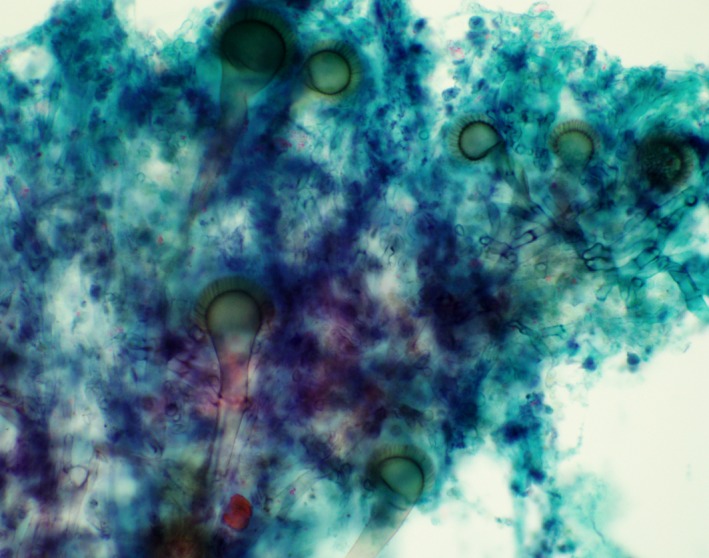
Smear preparation demonstrating conidiophores with globose conidial heads, a uniseriate vesicle with the metula covering the upper 2/3 of the vesicle (Papanicolaou stain)

**Figure 3:**
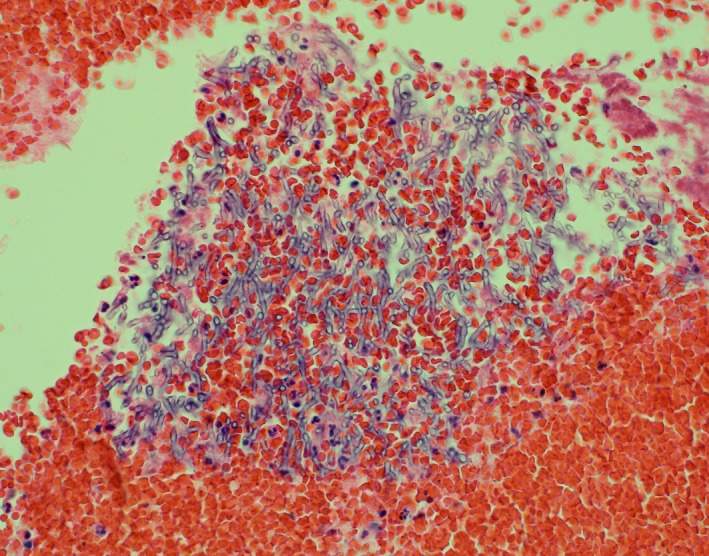
Cell block material demonstrating branching septate hyphae (Hematoxylin & Eosin Stain)

**Figure 4:**
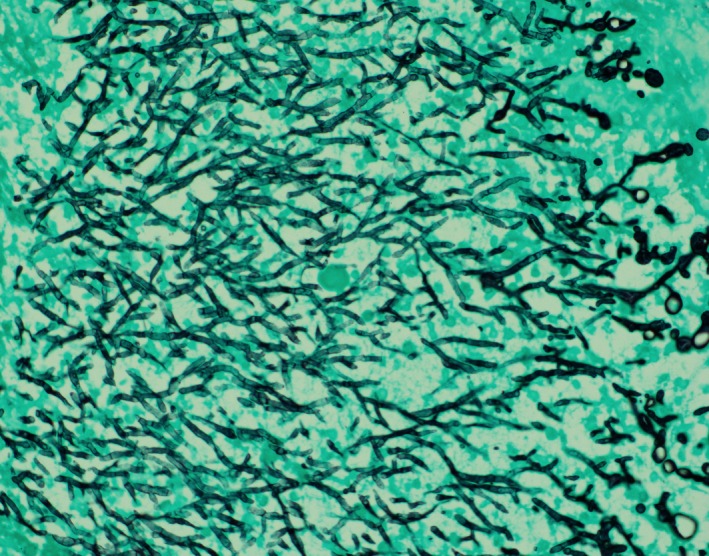
Cell block material demonstrating branching septate hyphae (GMS special stain)

The imaging findings in our case were concerning for a new primary lung malignancy with mediastinal lymph node involvement, given the patient's prior history of adenocarcinoma. However, cytological examination of lung aspirate revealed numerous fungal conidial and hyphal elements. The smears showed no malignant cells but demonstrated mucin strings and entangled hyphae in a background of inflammatory and necrotic debris (Figure 1). Numerous fungal hyphae were visible on all preparations with no evidence of malignancy. Fungal culture confirmed the presence of rare growth of *Aspergillus fumigatus*. The patient was offered treatment with voriconazole for suspected invasive disease but he refused treatment due to concerns of adverse effects. Subsequent clinical course and serial chest imaging findings over a period of 6 months revealed progression of primary lung cancer with metastasis. In our patient, the detection of *A. fumigatus* on fine needle aspiration cytology (FNAC) likely reflects colonization of the lung cavity and not invasive disease.

The differential diagnosis of a newly diagnosed cavitary lesion on chest imaging in an immunocompromised host is broad and includes malignancy, a variety of opportunistic infections, non‐infective granulomatous disease, and vascular infarcts.^1^, ^2^ A pulmonary cavity is defined as a gas‐filled lung area in the center of a nodule or area of consolidation and often detected by chest radiography or CT scan.^2^ Malignancy is the most frequent etiology of solitary cavitary nodule in the lung parenchyma. It may occur at any location and typically is round or irregular in appearance with a wall thickness >24 mm and associated perilesional consolidation.^1^, ^3^

Infectious etiology remains an important cause of cavitary lung disease. The microorganisms causing cavitary lesions in the lung may include bacteria (eg, *Streptococcus* species, *Staphylococcus aureus* including methicillin‐resistant* Staphylococcus aureus*,* Klebsiella* species,* anaerobes*); mycobacteria (eg, mycobacterium tuberculosis, atypical mycobacteria), and fungi (eg, *Aspergillus* species); and rarely parasites.^1^, ^2^ *Aspergillus fumigatus* is a ubiquitous hyaline mold which can cause serious and sometimes fatal infections in immunocompromised patients; in this setting, the organism causes disease predominantly in the lungs although dissemination to virtually any organ can occur.^4^, ^5^ In patients with altered lung function or individuals with an immunocompromised status, *Aspergillus* spp can cause a variety of pathological entities, including allergic bronchopulmonary aspergillosis, aspergilloma (occur in patients with underlying cavitary disease), chronic necrotizing aspergillosis, and invasive pulmonary aspergillosis, seen primarily in immunocompromised patients.^6^

Fine needle aspiration cytology can be quite helpful in distinguishing malignancy from infection.^7^ Fungal elements may be detected using routine stains as in our case. GMS special stain may be performed on cell block material. However, cavitary lung cancer, especially with cartilage tissue in the cavity wall can mimic aspergilloma.^8^ The diagnostic utility of fine needle aspiration cytology to detect a wide variety of opportunistic pulmonary infections in an immunocompromised host has been studied. FNAC techniques are safer, cost‐effective and provide rapid results.^7^ Uses of special stains to enhance cytomorphological changes in conjunction with microbiology culture results are critical to identify the specific microorganism.

## CONFLICT OF INTEREST

We have no conflict of interests to disclose.

## AUTHOR CONTRIBUTION

WL: was preparing and proofreading the manuscript. AKS: was treating the patient and preparing the manuscript. ZTS: is the corresponding author, was preparing the manuscript, and made revisions.
